# Sperm selection in IVF: the long and winding road from bench to bedside

**DOI:** 10.5935/1518-0557.20190081

**Published:** 2020

**Authors:** Moisa Lucia Pedrosa, Marcelo Horta Furtado, Márcia Cristina França Ferreira, Márcia Mendonça Carneiro

**Affiliations:** 1 Centro de Reprodução Humana Hospital MATER DEI, Belo Horizonte, MG, Brazil; 2 Departamento de Ginecologia e Obstetrícia e Obstetrícia da Faculdade de Medicina da UFMG, Belo Horizonte, MG, Brazil

**Keywords:** sperm selection, sperm sorting, noninvasive sperm evaluation, *in vitro* fertilization, intracytoplasmic sperm injection

## Abstract

Spermatozoa wage battle to conquer fertilization but the traits needed to succeed remain elusive. The natural advantageous qualities that enable only a few selected sperm cells to reach the site of fertilization remain unknown. Although *in vitro* fertilization (IVF) facilitates the job of spermatozoa, a universally acceptable means of sperm selection is yet to be developed. No objective or reliable sperm quality indicators have been established and sperm selection is, to a great extent, based on subjective qualitative evaluation. The best method for sperm selection in IVF presents several challenges: intrinsic sperm qualities cannot be evaluated and the ideal endpoint for these studies is debatable. An ideal method for sperm selection in ART should be noninvasive and cost-effective, and allow the identification of high-quality spermatozoa and yield better outcomes in terms of pregnancy and live birth rates. This narrative review included 85 papers and focused on the new available methods and technologies that might shed some light on sperm selection in IVF. It discusses the available data on microfluidic devices, omics profiling, micronuclei studies, sperm plasma membrane markers, and other techniques, such as Magnetic Activated Cell Sorting (MACS), Raman micro-spectroscopy, and artificial intelligence systems. The new techniques herein reviewed offer fresh approaches to an old problem, for which a definite solution has yet to cross the bridge from bench to IVF clinics around the world, since clinical usefulness and application remain unproven.

## INTRODUCTION

In ancient Sparta, selection criteria were rather strict and male babies were inspected by the Gerousia, the council of elders, and those judged unfit were left at Mount Taygetus to die. Spartans believed there was no place for those unable to fight for themselves. Though cruel, such strategy gave birth to a formidable army. 

Each sperm cell wages battle to conquer fertilization, but the traits needed to succeed remain elusive. The natural qualities that enable only a few selected sperm cells to reach the site of fertilization after going through obstacles inside the female reproductive system such as cervical mucus are unknown (Sakkas *et al*., 2015). *In vitro* fertilization (IVF) apparently demands much less effort from spermatozoa. Nevertheless, decades of research have not found a universally accepted sperm selection procedure (Yetunde & Vasiliki, 2013; McDowell *et al*., 2014).

Traditionally, the World Health Organization (WHO, 2010) thresholds are used to evaluate semen quality, which unfortunately does not correlate well with sperm quality and/or function. The development of assisted reproductive technologies (ART) opened parenthood opportunities. However, success rates are unfortunately not satisfactory, and many couples remain childless even after several attempts (Toner *et al*., 2016). Intracytoplasmic sperm injection (ICSI) introduced by Palermo *et al*. (1993) represented a major breakthrough in male infertility as it made pregnancy possible even in the most severe cases until then considered untreatable. In ICSI, one “normal motile sperm” is injected into the oocyte yielding high fertilization and pregnancy rates (Palermo *et al*., 1993). Unfortunately, no objective and reliable sperm quality indicators have been established so far and sperm selection remains based on subjective qualitative evaluations performed by experienced embryologists. Learning more about sperm quality and establishing criteria to select the fittest spermatozoa is crucial if we are to improve ART success rates.

Establishing the best method for sperm selection in ART presents several challenges. Techniques used to prepare sperm for ART do not seem to select the most suitable sperm for fertilization, since they cannot evaluate intrinsic sperm traits that may be fundamental for fertilization (Yetunde & Vasiliki, 2013; Sakkas *et al*., 2015). A Cochrane review looked into advanced selection methods for ART including selection according to surface charge; sperm apoptosis; sperm birefringence; ability to bind to hyaluronic acid; and sperm morphology under ultra-high magnification. Unfortunately, it could not find grounds for the application of such techniques in clinical practice (McDowell *et al*., 2014). To make matters worse, there is the question of how to measure method effectiveness. If sperm fitness were measured in terms of ability of achieving pregnancy, for example, a variety of factors involving the oocyte, the sperm, and the uterus might interfere in the final outcome (Berkovitz *et al*., 2006; Simopoulou *et al*., 2016). An ideal method for sperm selection in ART should be noninvasive and cost-effective, and allow the identification of high-quality spermatozoa and yield better outcomes in terms of pregnancy and live birth rates (Rappa *et al*., 2016).

We therefore set out to find the published evidence on newly available methods and technologies that might shed some light on the matter of sperm selection in ART and compile the information into a narrative review. We aimed at finding novel techniques for sperm selection in IVF that might eventually cross the bridge from bench to bedside.

## MATERIAL AND METHODS

We searched for papers available on PubMed up to April 2019 using the following keywords: sperm selection, sperm sorting, noninvasive sperm evaluation, *in vitro* fertilization (IVF) and intracytoplasmic sperm injection (ICSI).

No language or time restriction was applied, but only articles pertaining to humans were included. The first step in the selection process revolved around reading the titles of the papers found in the search and the removal of studies not relevant to the matter at hand. The abstracts of the remaining publications were read in the second step of the selection process. We focused on studies performed in humans in the area of ART that published outcomes in terms of fertilization, and pregnancy and/or live birth rates whenever possible.

We left out Sperm DNA fragmentation, which has been associated with adverse outcomes in IVF including reduced fertilization and pregnancy rates, poor embryo quality, and increased miscarriage rates, as it has been extensively studied without leading to valid practical conclusions (Cissen *et al*., 2016). Unfortunately, published studies argue against the routine use of sperm DNA fragmentation tests in couples undergoing IVF, since it does not predict pregnancy rates or aid in the choice of treatment (Evenson, 2016; Simon *et al*., 2017). Other sperm selection techniques such as intracytoplasmic morphologically selected sperm injection, hyaluronic binding, polarized light microscopy, and annexin V agent identification for comparing sperm cells and their chromatin integrity were also excluded form our review as they have already been extensively reviewed elsewhere (Simopoulou *et al*., 2016).

## RESULTS

Our initial PUBMED search revealed the following results:

- Sperm selection, IVF: n=494
- Sperm selection, ICSI: n=622
- Noninvasive Sperm selection, IVF: n=22
- Noninvasive Sperm selection, ICSI: n=25
- Sperm sorting, IVF: n=22
- Sperm sorting, ICSI: n=25

The search proved difficult, since many of the publications touched upon other aspects of sperm preparation in IVF and did not address the specific question we asked on the subject of sperm selection in IVF focusing on results in terms of fertilization, pregnancy and/or live birth rates whenever possible. Many publications consisted of reviews and described technical aspects without showing clinical application. Therefore, in the end we had ende 73 papers discussing the use of the new techniques described below.

### Microfluidic devices and sperm selection

A number of biophysical factors are important regulators of gamete and embryo function, but improved understanding of the physical forces involved in the processes of human reproduction requires novel experimental platforms. In order to bridge this gap, engineers are building tools to control mechanical factors with improved precision and throughput, thereby enabling biological investigation of mechanics-driven function in an attempt to improve understanding and IVF results (Carneiro *et al*., 2015).

In the human body, there are small channels in the human body containing moving fluid that make up most of the conduits. Microfluidic devices act as a physiological platform to recreate the channels and fluid flows in a living organism. These tiny devices have precise dimensions that allow control over the biophysical and biochemical environment at a quantitative level, while the results of the experiments are visualized using optical microscopy (Zhang *et al*., 2011; Smith & Takayama, 2017).

Microfluidic technology offers the possibility to model sperm journey throughout the female reproductive tract as the male gamete swims through these biophysical and biochemical environments, while allowing evaluation of sperm motility dynamics at a quantitative level (Suarez & Wu, 2017). Thus, microfluidics may help develop new techniques for gamete selection with minimum damage. The majority of such devices work by improving the swim-up method, resulting in a highly selected subpopulation of spermatozoa in sufficient numbers for IVF. Other devices use physical markers such as morphology to identify the best sperm cells. Microfluidics can also be used in combination with other techniques such as Raman Spectroscopy (Samuel *et al*., 2018).

Xie *et al*. (2010) reported improved proportions of motile sperm after using a micro device consisting of a straight channel connected with a bibranch channel mimicking the female reproductive system. No clinical ART data was reported. Other studies revealed that the use of microfluidic devices resulted in the selection of sperm with reduced DNA damage in comparison to the swim-up technique (Kishi *et al*., 2015; Shirota *et al*., 2016). The group of men studied, however, was small, heterogeneous, and included both fertile and infertile individuals, thus preventing solid conclusions. Quinn *et al*. (2018) used discarded, non-clinical semen samples in an attempt to find whether microfluidic sorting might improve the selection of sperm with lower DNA fragmentation over standard density-gradient centrifugation. DNA fragmentation levels after microfluidic sorting were almost undetectable, but standard processing did not increase fragmentation either.

None of the studies using microfluidics devices in IVF cycles reported outcomes in terms of fertilization or pregnancy rates. Data from animal studies, however, seem encouraging. Further studies with more participants on the clinical use of microfluidics devices are needed so as to evaluate the safety and usefulness of such devices. In addition, numerous types of devices are available, with not a single one successfully applied in clinical setting so far (Suarez & Wu, 2017).

### Omics and sperm selection

Proteomics is a promising, fast-developing technology-driven field that might provide important contributions to ART by allowing the identification of many potential biomarkers as we elucidate physiological mechanisms vital for oocyte fertilization, embryo development, and improved live birth rates in this setting (Sakkas *et al*., 2015; Kosteria *et al*., 2017). Unfortunately, only a handful of reports have been published so far and they did not focus on sperm selection for IVF.

Azpiazu *et al*. (2014) published a case-control study involving 31 men with normozoospermic sperm and their partners who underwent IVF with successful fertilization divided into two groups: 16 couples unable to achieve pregnancy after IVF and 15 that achieved pregnancy after IVF. Proteomic analysis revealed that the groups showed differences in the levels of at least 66 proteins. Zhu *et al*. (2013), on the other hand, found 21 proteins that were differentially expressed (>1.2-fold) in men whose sperm resulted in clinical pregnancies in comparison to individuals unable to achieve clinical pregnancy. Other authors have also reported these differences in similar circumstances (Pixton *et al*., 2004; Frapsauce *et al*., 2009; 2014). The potential role of these differences in IVF failure in terms of fertilization and pregnancy rates remains to be established (Holland & Ohlendieck, 2015).

Metabolomics involves the identification of metabolites, small molecules that represent the final product of the interaction between genetics and the environment, and might thus produce a more accurate reflection of physiological and pathological events within an organism (Courant *et al*., 2013). Metabolomics has been used to identify serum fingerprints in Danish men with low, intermediate, and high sperm concentrations. Results showed significant differences among the three groups (Courant *et al*., 2013).

A differentiated serum metabolomic profiling has also been described in non-obstructive azoospermic men in comparison to healthy control subjects. Results identified 24 metabolites involved in crucial steps for spermatogenesis, such as energy production, oxidative stress and cell apoptosis (Zhang *et al*., 2017).

Zhao *et al*. (2018) published, for the first time, the metabolic profile of human sperm cells using an untargeted platform based on gas chromatography-mass spectrometry (GC-MS). Normal healthy man and individuals with idiopathic asthenozoospermia were evaluated. Twenty-seven metabolites showed reduced levels in the idiopathic asthenozoospermia group compared with the normozoospermic group, while six were increased in idiopathic asthenozoospermia.

Despite the various published papers and their level of sophistication, the OMICS techniques have not been used to select good quality sperm in IVF. The techniques used either to screen for proteomic or metabolomic fingerprints vary and reproducible results are still a promise rather than a fact, and clear diagnostic and/or quality markers remain to be discovered. 

### Micronucleus test and sperm selection

Micronuclei (MN) in mammalian cells are produced during anaphase 1 in mitosis or meiosis as chromosomes separate, and the either whole chromosomes or fragments lagging behind possibly due to DNA damage can be observed in cytoplasm close to the cell nucleus as small nucleus-like particles. MN development may be due to various factors such as acquired or inherited genetic alterations, deficiency of micronutrients which act as co-factors in DNA metabolism, or exposure to genotoxicants (Fenech, 2011; Fenech & Bonassi, 2011).

MN have been associated with serious genetic alterations in daughter cells and their development results in abnormal gene expression and diminished proliferative potential. Indeed, increased MN frequency is related to cytotoxicity, cell development arrest, and death (Kirkland, 2010). MN production takes place in reproductive tissues such as male germ cells, placenta, and the embryo, therefore resulting in adverse consequences on fertility and pregnancy (Kamiguchi *et al*., 1991; Trková *et al*., 2000; Özden et al., 2014).

Some studies advocate the use of the MN assay to investigate the association between DNA damage and reproductive failure in humans (Kamiguchi *et al*., 1991; Trková *et al*., 2000; Fenech, 2011; Özden et al., 2014). Trková *et al*. (2000) found increased micronucleus frequency in the lymphocytes of couples with infertility or two or more spontaneous miscarriages, suggesting a possible link between chromosomal instability and reproductive failure. However, it was not possible to define a male role in the miscarriages neither was MN tested in the couple’s lymphocytes, and results were analyzed taking into consideration male and female factors.

Published studies report on MN assay in lymphocytes, since there is no technique developed to assess MN directly in human sperm. Sperm exhibits an extremely compacted DNA and harbors almost no cytoplasm where MN might be detected (Fenech, 2011). The debate remains as to whether MN assayed in lymphocytes reflects DNA damage in other cells such as spermatozoa (Fenech, 2011). Kamiguchi *et al*. (1991) have described the use of MN testing to assess radiation-induced chromosomal damage in human spermatozoa. Unfortunately, no other studies were performed using the technique to assess sperm damage or sperm quality.

Milošević-Djordjević *et al*. (2012) investigated chromosomal instability using the MN test in blood lymphocytes of patients with reproductive failure taking into consideration age, smoking habits, gender, miscarriages, and semen analysis. Subjects with reproductive failure presented with increased baseline MN frequency probably related to increased chromosomal damage.

Our literature search revealed that MN testing is a promising tool, although more research is needed before it is used in clinical practice. MN assaying, however, might turn into a practical way to assess sperm DNA damage once standard protocols have been developed and validated (Fenech, 2011).

### Sperm plasma membrane marker

Although the role of plasma membrane markers is not new, the subject is still understudied. As a matter of fact, the two most used tests nowadays are both based on selection of sperm according to membrane integrity. The first uses magnetic activated cell sorting (MACS) with colloidal super-paramagnetic microbeads conjugated with annexin V. The most widely evaluated sperm selection technique selects sperm cells that bind to hyaluronic acid (HA), the main component of the extracellular matrix of the *cumulus-oophorus*. Many other plasma membrane characteristics and markers have been evaluated and some are detailed below.

Electrophoresis and electronegative charge were found to produce sperm populations enriched in DNA-intact spermatozoa. (Ainsworth *et al*., 2005; Chan *et al*., 2006; Razavi *et al*., 2010). The main source of negative charge on the sperm plasma membrane has been attributed to a specific GPI-anchored glycoprotein, CD52 (Schröter *et al*., 1999), which carries highly-sialylated-polylactosamine-containing carbohydrate chains. Increased sperm surface negative charge is a correlate of sperm maturation in the epididymis, and therefore this selection method is likely to act as a filter for mature versus immature spermatozoa. Indeed, the three methods of separation by sperm plasma membrane electronegative charge - the Zeta test (Chan *et al*., 2006; Kam *et al*., 2007), electrophoretic sperm separation (Ainsworth *et al*., 2005; 2007) and more recently micro-electrophoresis (Simon *et al*., 2015) - were shown to isolate sperm cells that are mature, viable, motile, morphologically normal, nonapoptotic, and displaying low levels of DNA damage (Ainsworth *et al*., 2007; 2011;Chan *et al*., 2006; Kam *et al*., 2007; Nasr-Esfahani *et al*., 2009; Khajavi *et al*., 2009; Razavi *et al*., 2010; Simon *et al*., 2015).

Ubiquitin is a small chaperone molecule known mainly from post-translational modifications called ubiquitination. Ubiquitin as a quality control marker is secreted by the epididymal epithelium to eliminate defective spermatozoa by subsequent phagocytosis (Da Silva & Barton, 2016; Richburg *et al*., 2014; Sutovsky *et al*., 2001a). In spite of this mechanism, some of the defective spermatozoa tagged by extracellular/cell surface ubiquitination are carried over into the ejaculate. Thus, ubiquitin might be used as an appropriate sperm marker (Sutovsky *et al*., 2001a;b).

Opsins are a family of G-protein coupled receptors (GPCRs) thought to act as thermosensors for sperm thermotaxis (Pérez-Cerezales *et al*., 2015). Recently, Pérez-Cerezales *et al*. (2018) in a study using mice, found a link between a physiological characteristic of sperm - the capacity to migrate in a temperature gradient - and the quality of its genetic content. The results of the study pointed to thermotaxis as both a guidance mechanism and a means of selecting high quality mammalian spermatozoa. In mice, its use dramatically improved the efficiency of ICSI giving rise to high quality embryos. Sperm thermotaxis has a promising role as a selection method for basic sperm studies and ART.

Chemotaxis is another important way to guide the sperm to the oocyte. Sperm chemotaxis is modulated by progesterone through its receptor, CatSper (Arnoult *et al*., 2011; Lishko *et al*., 2011; Strünker *et al*., 2011), and via chemokine-receptor interactions involving factors produced by the oocytes, granulosa cells, and endometrial cells. Thirty percent of live human spermatozoa express CXCR4 (chemokine CXC motif receptor 4) (Kim *et al*., 1999; Zuccarello *et al*., 2011), meaning that 70% of spermatozoa might be unresponsive to chemical signals emanating from granulosa cells and oocytes. Another common chemokine receptor, CCR6, was not detected in every cell. The existence of this chemotactic interaction mechanism, together with differential expression of specific receptors among spermatozoa, is consistent with the concept of a molecular passport for spermatozoa based on between-sperm differences.

Sperm membrane glycoproteins have a direct role in sperm-egg adhesion and fusion during the fertilization process (Bronson *et al*., 1999; Wolfsberg *et al*., 1993). Some of the main potential biomarkers involved in zona pellucida penetration, sperm binding, and oocyte fertilization are heat shock protein (HSPA2), serum amyloid P compound (SAP), cysteine-rich secretory proteins (CRISP), fertilin β (Fβ), PH-20, DJ-1, and epididymis P34H protein (Fusi & Bronson, 1992; Bohring & Krause, 2003; Kiernan *et al*., 2004; Snell & White, 1996; Wagenfeld *et al*., 2000). Three of them - HSPA2, DJ-1, and SAP - are responsible for fixing the DNA strand breaks, replacing protamine through nuclear compaction, and eliminating the cytoplasm in the last stages of sperm maturation in human testes (An *et al*., 2011). Moreover, DJ-1 has a major role in androgen receptor-dependent transcriptional activity and oxidative stress (Saylan & Duman, 2016; Bonifati *et al*., 2003; Mitsumoto & Nakagawa, 2001; Taira *et al*., 2004; Takahashi *et al*., 2001). SAP in humans has physiological and pathological roles in inflammation, immunity, and apoptosis (Bickerstaff *et al*., 1999). Therefore, it is suggested that HSPA2, DJ-1, and SAP might be a potential method to select sperm with the lowest level of chromatin damages in ART. HSPA2 is also induced in response to the presence of environmental agents such as stress, air pollution, and oxidative stress (Hahn & Li, 1982), so this might be a useful method to select sperm with less damage from reactive oxygen and nitrogen species.

### Other strategies under investigation

Flow cytometric sorting has been used to select spermatozoa with low DNA fragmentation rates for IVF. Results show that flow cytometric sorting selected significantly fewer spermatozoa with fragmented DNA when compared to the conventional swim-up technique (Ribeiro *et al*., 2013). Questions related to the safety of the process have been raised concerning the mechanical damage that sorting may inflict upon spermatozoa as they pass through the microfluidic channels, which may potentially adversely affect sperm viability, motility, and velocity (Caroppo, 2013).

Magnetic Activated Cell Sorting (MACS) has been described as a sperm selection technique in IVF. Results of a systematic review and meta-analysis of prospective randomized trials showed statistically significant differences in pregnancy rates when MACS was used to select sperm compared with conventional techniques (either density gradient centrifugation and swim-up), but implantation rates were unaffected. Live birth rates were reported (Gil *et al*., 2013). The ability of MACS to effectively select sperm with reduced DNA fragmentation has been recently questioned (Martínez *et al*., 2018), and although its use did not improve live birth rates, fewer miscarriages were recorded (Sánchez-Martín *et al*., 2017).

Raman micro-spectroscopy has been described as an innovative method to assess sperm features with potential use as a non-invasive selection method, since it may aid in the identification of sample variations without external labels or extensive preparation tool (Liu *et al*., 2014). The technique has been used in forensic medicine to effectively identify semen. Further uses include the identification of chemical signatures related to various sperm functions, a promising application in sperm assessment in IVF (Mallidis *et al*., 2014). Concerns related to the possible effects of laser on spermatozoa still remain and may hamper the use of the technique.

Research has so far presented controversial data on sperm evaluation, and except for nuclear DNA, the identification and assignment of spectral bands in Raman-profiles to the different sperm regions remains to be established (Amaral *et al*., 2018). Raman microspectroscopy is a promising tool in the assessment of male fertility, but studies are, unfortunately, in their infancy, which may delay its potential use in IVF.

Artificial intelligence (AI) systems have been used to categorize oocytes and embryos and reliably identify perfect oocytes and embryos that lead to pregnancy. The use of such approaches might result in objective, automatic, non-invasive oocyte or embryo evaluation (Manna *et al*., 2013). Similar systems using computational techniques have been applied to the study of sperm locomotion and in mapping trajectories and calculating numerous motility parameters (Daloglu & Ozcan, 2017). Our search for studies on the use of AI for sperm selection found no matches.

## CONCLUSION

Available published evidence does not show a clear advantage of using advanced techniques for sperm selection in IVF, since they have not been correlated with improved outcomes in terms of fertilization, implantation, or live birth. In addition, these techniques involve extensive sperm manipulation and possible exposure to chemicals and non-physiological environments that might adversely affect sperm DNA and result in disrupted embryo development (Said & Land, 2011). As the selection of spermatozoa with healthy DNA should be the primary aim of such techniques, a word of caution is worth consideration before crossing the bridge from bench to bedside. Unfortunately, the techniques discussed herein do not offer immediate hope when sperm selection in IVF is concerned. We provided an analysis of novel techniques with their strengths and limitations, a topic that currently poses many questions to reproductive medicine specialists worldwide. An ideal method for sperm selection in IVF should be noninvasive and cost-effective, and allow the identification of high quality spermatozoa and yield better outcomes in terms of pregnancy and live birth rates.

Sperm cells are rather complex and sophisticated. They must be as fit as Spartan soldiers in order to perform highly specialized functions and achieve fertilization. Unfortunately, despite decades of research, the currently available sperm function and selection tests have failed to offer the long expected improvement in ART success rates. Failure might be attributed to the complexity of spermatozoa and the battles these cells must wage in order to achieve fertilization and pregnancy. The strategies sperm cells use to win the battles they face while traveling the female reproductive system remain elusive. The new techniques reviewed herein offer fresh approaches to an old problem, for which a definite solution has yet to cross the bridge from bench to IVF clinics around the world. After all, in the wise words of Lennon, McCartney et al., this is a long and winding road.
